# Factors that impacted mobile-payment adoption in China during the COVID-19 pandemic

**DOI:** 10.1016/j.heliyon.2023.e16197

**Published:** 2023-05-12

**Authors:** Kamal Abubker Abrahim Sleiman, Lan Juanli, Hong Zhen Lei, Wenge Rong, Wang Yubo, Shunhang Li, Jingyi Cheng, Fouzia Amin

**Affiliations:** a School of Economics and Management, Yan'an University, Yan’an City, 716000, China; b International Business School, Shaanxi Normal University, Xi’an, 710062, China; c School of Computer Science and Engineering, Beihang University, Beijing, 100191, China; d Affiliated Hospital, Yan'an University, Yan'an City, 716000, China; e National Defence University, Sector E-9, Islamabad, Pakistan

**Keywords:** Mpayment, C-19, UTAUT, Social distancing, Behavioral intention

## Abstract

The Unified Theory of Acceptance and Use of Technology (UTAUT) is a potential paradigm for explaining technology adoption and can be applied to a wide range of scenarios. During the COVID-19 (C-19) outbreak in China, mobile-payment platforms (Mpayment) were used extensively in everyday life because they allowed people to avoid direct and indirect connections during transactions, adhere to social-distancing guidelines, and support social-economic stabilization. By exploring the technological and psychological variables that influenced user Mpayment-adoption intentions during the C-19 pandemic, this study broadens the literature on technology adoption in emergency circumstances and expands the UTAUT. A total of 593 complete samples were collected online, with SPSS used for data analysis. The empirical findings reveal that performance expectancy, trust, perceived security, and social influence all had a significant influence on Mpayment acceptance during the C-19 outbreak, with social distancing having the greatest impact, followed by fear of C-19. Interestingly, perceived-effort expectancy had a negative influence on payment acceptance. These findings suggest that future studies should apply the expanded model to different countries and areas to investigate the impact of the C-19 pandemic on Mpayment acceptance.

## 1 Introduction

A dramatic rise in the use of smartphones has changed people's everyday lives, particularly in relation to financial transactions. Recently, mobile-payment platforms (Mpayment) have been incorporated into a wide and varied range of businesses. According to WorldPay research, Mpayments accounted for 22% of worldwide point-of-sale spending in 2019, with that proportion predicted to increase to 29.6% by 2023. Furthermore, China's widespread use of Mpayments (e.g., Alipay and WeChat Pay) at the point of sale via QR codes accounted for nearly half (48%) of all point-of-sale payments in 2019. Several prior studies have explored the Mpayment-adoption perceptions in various settings [1,2].

The C-19 pandemic began in December 2019 and spread rapidly worldwide. To date, the World Health Organization has reported 66,243,918 affirmed COVID-19 (C-19) cases and 1,528,984 deaths globally [3]. Tang et al. [4] strongly recommended restricting contact between people and maintaining social distancing practices, given the high risk of C-19 transmission. Contactless payments, while helping to meet consumer expectations of assistance during the use of payment systems, can also protect consumers and ensure their safety. Consequently, the use of payments in China expanded dramatically during the C-19 pandemic. According to *China Banking and Insurance News* (2020), during the C-19 pandemic, the number of Mpayment transactions in China rose to 22.4 million in the first quarter of 2020, an increase of 187% from 2019 [5]. In the ATM marketplace, C-19 prompted many individuals to adopt digital-payment practices, including contactless transactions, remote-teller assistance/videos, and mobile-phone incorporation [6]. According to a 2020 CNNIC report, the percentage of mobile-device users making payments in China increased from 73.5% in June 2019 to 85.3% in March 2020—and then to 86.0% in June 2020, confirming that Mpayments help to keep individual and company transactions running during crisis situations [7]. In recent years, customer and business payment patterns have shifted away from traditional face-to-face transactions towards contactless payments. During the pandemic, mobile money transactions have enabled many businesses to survive and helped to develop the social economy. This raises the following question: What factors make customers more likely to use Mpayments during a pandemic or epidemic? Understanding customer actions during the C-19 pandemic can help researchers and stakeholders examine IT acceptance in crisis situations and design appropriate business strategies. The Unified Theory of Acceptance and Use of Technology (UTAUT) can be used to assess consumer intentions, based on technological perceptions, although it cannot assess fully the impact of other people's judgments.

The current study explores factors that impacted Mpayment acceptance from the customer's viewpoint during the C-19 pandemic. The UTAUT is considered the most well-developed, up-to-date, and relevant technology-acceptance model (TAM) [8]. The UTAUT model, created to investigate user behavior and provide a unified picture of IT [9], was tested by Smith et al. [9], in a longitudinal study and found to be accurate. UTAUT is adopted in the present study because it is more comprehensive than earlier theories of technological acceptance and provides greater explanatory power. Researchers have studied the model over time by incorporating various factors to enable a better understanding of technology uptake in relation to situational aspects of the research area [10]. Here, integrating social distancing and fear of COVID-19 into the model provides a better understanding of users' behavioral intentions toward technology uptake during the recent pandemic, which has changed user behaviors. The UTAUT model is suitable for evaluating payment-acceptance systems because it is the most modern and up-to-date technology-acceptance theory available and widely accepted by researchers [8].

A review of the recent literature reveals that very little is presently known about the way in which the COVID-19 pandemic and social distancing have impacted the consumer adoption of cashless-payment methods. This study aims to determine the reasons for shifts in customer-payment behavior observed throughout the epidemic. In doing so, it fills gaps in the literature on customers' payment behavior and habits during the COVID-19 pandemic.

This paper includes the following sections: 2) a review of the literature; 3) theoretical and research hypotheses; 4) research methodologies; 5) data analysis and discussion; 6) theoretical and practical implications; 7) limitations and future research; and 8) conclusions.

## 2 Related work

Compared to traditional payment methods, Mpayment has become increasingly popular in China, allowing customers to make a wide range of transactions without time or location restrictions [11,12]. Mpayment is a critical component of mobile commerce [13] and a method of making payments for services, commodities, and bills; it can also assist with other forms of communication [14]. Mpayment has been described as a technological innovation that reflects a conventional method of payment: “any payment where a mobile device is used to initiate, authorize, and confirm an exchange of financial value in return for goods and services” [15,16]. The ultimate objective of Mpayment is to control the time and location of traditional and cash-centric payments in general [17]. Chinese payment services are supported by third-party payment systems, such as Alipay and WeChat Pay, which facilitate payments [17,18].

As Mpayment offers convenience and security, an innovative business climate has emerged as a result of its widespread adoption. Financial transactions can now be conducted anytime, anywhere, and by anyone, creating vast market opportunities in a diverse range of scenarios, particularly during pandemics [12].

TAM is derived from the Theory of Reasoned Action (TRA), which was created to illustrate the use of human technology [19]. The model demonstrates that perceived usefulness (PU) and perceived ease of use (PEOU) are the determinants of consumer intention to use a platform. PU refers to “the degree to which a person believes that using a particular system would enhance his/her job performance,” while PEOU is the degree to which “a person believes that using the system will be free of effort” [20]. These two advantages contribute to a positive mindset and the desire to use the platform in question. The model can also be applied to different settings. Since its emergence, modified TAMs have been proposed in response to new technologies [21,22].

Most technology models have some limitations. Although TAM is a remarkable model in the field of technology acceptance and adoption, it does “exclude the possibility of influence from institutional, social, and personnel control factors” [23]. As the model cannot anticipate people's general adoption needs, it must be re-evaluated regularly. TAM constructs cannot capture fully the unique impact of technical and use-related background factors that can alter its acceptance [24,25]. Many adjustments have been made to the original TAM, based on these findings. The most powerful new model is UTAUT, which incorporates common conceptual and practical features from eight models [26]. UTAUT offers a straightforward look at the way in which behavioral-intention factors change over time. It suggests using three dominant direct intention-to-use factors, namely, “performance expectancy, effort expectancy, and social influence,” alongside the direct usage-behavior factors of “intention and facilitating conditions.” Despite these advances, both TAM and UTAUT have faced criticism [27].

As a worldwide pandemic, COVID-19 has significantly impacted people's everyday lives and the international economy [4]. Human contact has been shown to have a clear and considerable impact on the risk and rate of coronavirus infection. The contactless function that forms a common element in all Mpayment systems can support, protect, and preserve user transaction experiences [28]. As a consequence of regulations imposed by the Chinese government to minimize direct contact and maintain social distancing during the pandemic, Mpayments have been widely used and accepted for their contactless characteristics and reliability. Individuals have been encouraged to develop a positive understanding and perception of safety when using Mpayment as their primary payment system in order to reduce the danger of virus transmission, ensure personal safety, and support the social economy [5].

## 3 Theoretical model and research hypothesis

Venkatesh and Morris [9] developed UTAUT to determine individual behavioral intentions to adopt a new technological system. UTAUT has been used in a wide range of technology-adoption contexts, with additional factors added to explain the behavioral intentions of users [3]. For example, Ref. [29] combined security-related factors with the UTAUT model and showed that security and trust had a significant impact on user intentions to use NFC payments in restaurants. Moreover, Ref. [30] extended the UTAUT model by using the additional factors of perceived trust and satisfaction to assess Mcommerce usage intentions.

**Fig. 1:**
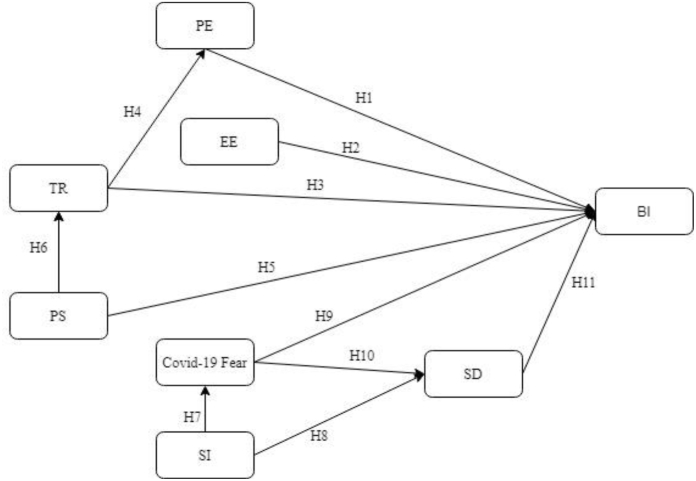
The study model.

This study extends UTAUT by adding some new components, such as social distancing and fear of C-19, to the basic model. These components also include performance expectancy, effort expectancy, social influence, and facilitating conditions (trust and security). This study expects the model to be used to investigate the acceptability of Mpayments during the C-19 pandemic. The authors have therefore applied and extended the model presented in Fig. 1 to examine the impact of social distancing and fear of C-19 on Mpayment behavioral intentions.

### 3.1 Performance expectancy

Performance expectancy is defined as the users' view of how an IS will help them complete a task and perform at work [9,31]. It can also be described as the extent to which individuals comprehend a system's ability to help them make better payments. The term implies that users will be able to implement a system if they comprehend how it can improve their efficiency. Performance is achieved using attributes related to the system’s efficiency, speed, and precision in performing a task [32]. In relation to payment acceptance, performance expectancy has a beneficial effect on user adoption intentions in various circumstances [12]. Users have been particularly concerned about payment efficiency and accuracy during the C-19 pandemic. As a result of the C-19 pandemic, completely new variables have entered the process through which consumers and businesses can minimize the use of cash in their financial activities [1]. When it comes to payment adoption, performance expectancy has a significant and positive impact on user adoption intentions in a wide variety of circumstances. Based on this argument, the following hypothesis is proposed:

H1 Performance expectancy has positively affected the behavioral intention to adopt Mpayment during the C-19 pandemic.

### 3.2 Effort expectancy

According to UTAUT, effort expectancy is “*the degree of ease associated with the system’s use”* [9]. User opinions about adopting Mpayment are impacted by the level of effort required [12]. In fact, effort expectancy typically has a greater influence on user attitudes than performance expectancy [8,31]. Effort expectancy has been shown to be the most significant element affecting consumer intentions to use NFC Mpayments for public transportation [33]. Based on these findings, we propose the following hypothesis:

H2 Effort expectancy has positively influenced the behavioral intention to adopt Mpayments during the C-19 pandemic.

### 3.3 Perceived trust

*Trust is* defined as the psychological expectation of having a reliable partner who will not be opportunistic [34]. It can also be defined as a service provider's commitment and willingness to satisfy consumer expectations. Trust is an important feature because it lessens the uncertainty, fear, and concern associated with using a service [35]. According to the Theory of Planned Behavior (TPB), trust influences users' behavioral intentions [36]; it also has a direct association with the propensity to purchase from online suppliers [37,38]. Trust has been integrated into the TAM to analyze the behavior of Mpayment consumers [39]. In recent years, the C-19 pandemic has increased the fear and social pressure associated with people’s daily transactions. Due to the trustworthiness of Mpayment platforms, users are more likely to make contactless Mpayments in place of traditional payments [30]. After the adoption of a trust-based adoption model, trust has been shown to have strong direct and indirect effects on the behavioral intention to use Mpayments [28]. It has been confirmed as an additional UTAUT factor that improves performance expectancy and has a favorable impact on users' behavioral intentions to use Mpayments [8]. The following hypotheses are therefore proposed.

H3 perceived trust has positively affected the behavioral intention to adopt Mpayment during the C-19 pandemic.

H4 perceived trust has positively affected performance expectancy, leading to the adoption of Mpayments during the C-19 pandemic.

### 3.4 Perceived security

In the context of Mpayment methods, perceived security is defined as “the degree to which the user believes that using a technology Mpayment process will be secure” [40]. Security has been shown to be a vital feature for online consumers [41]. Previous research has demonstrated that user perceptions of security are a crucial element, influencing whether they choose to use Mpayments [33]. Furthermore, perceived security promotes user trust by shielding consumers from transaction uncertainties [42]. As a result of the perceived security of Mpayments, user trust increased throughout the C-19 pandemic. For this reason, we have added “security” to UTAUT and propose the following hypotheses:

H4. Perceived security has positively affected the behavioral intention to adopt Mpayments during the C-19 pandemic.

H5 Perceived security has positively affected trust, increasing the adoption of Mpayments during the C-19 pandemic.

### 3.5 Social influence

According to UTAUT, social influence is defined as “the extent to which an individual believes that he/she should utilize the new system” [9]. During the C-19 pandemic, references and recommendations from relevant people became even more influential in guiding individuals to make decisions and take appropriate actions. Previous studies have examined social influence extensively in different situations to determine its impact on the intention to use Mpayments [43,44]. According to Al-Qudah et al., [45], social influence has a considerable effect on the behavioral intention to use mobile payments. People are socially influenced because highly contagious diseases have a relatively high mortality rate, which naturally increases fear. In the case of C-19, the virus spreads through interactions with people infected with the disease [3]. To control this highly infectious virus, governments worldwide issued policies, such as social distancing, to reduce the spread of C-19 [46]. Based on this, we propose the following hypotheses.

H7 Social influence increases fear of C-19, prompting people to adopt Mpayment during the C-19 pandemic

H8 Social influence prompts people to adhere to social-distancing guidelines by adopting Mpayment during C-19 pandemic.

### 3.6 Fear of Covid-19

In December 2019, a new coronavirus disease emerged, with the first case discovered in China and then transmitted all around the country. Although studies have shown that fear can be a positive factor when an actual danger exists, fear of C-19 has become chronic and stressful for many people [47]. The C-19 pandemic has prompted changes in various economic sectors and corporate strategies, impacting customer behavior in many different sectors, including everyday payment patterns [48]. C-19 has disrupted people’s normal patterns of response to environmental cues, while simultaneously driving non-standard behavior. Despite the lack of any extra financial incentives, cashless payment systems have increasingly been adopted during this period. In comparison to the level of cashless-payment adoption that existed before 2019, the rate at which societies are moving away from cash has accelerated since the pandemic began [49,50]. Social constraints are therefore needed to limit the spread of C-19. To achieve success, it is crucial to remember that social distancing should not be equated with social disconnection [51]. According to recent studies, the fear of spreading infection has significantly influenced people’s willingness to abandon cash and motivated them to adopt contactless payment technologies. Recent research has also revealed that the C-19 pandemic has played a significant role in altering public perceptions of and support for branchless banking [52]. We therefore suggest the following hypothesis:

H9 The fear of C-19 has encouraged Mpayment adoption during the C-19 pandemic.

H10 The fear of C-19 has encouraged social distancing during the C-19 pandemic.

### 3.7 Social distancing

Social distancing has been implemented for the general population to prevent the spread of C-19 [51]. Social-distancing strategies can be useful against pandemics but are economically damaging [53]. During the pandemic, social distancing, aided by contactless payments, has allowed consumers to function quite normally. The characteristics of mobile and contactless payment allow for social distancing by eliminating interactions with currency. Customers can avoid touching terminals via contactless payments (such as smartphone payments), while also keeping their distance from merchants. Unlike cash, which is generally held in the vendor's hand, digital-payment devices are occasionally set up far from the seller [1,54]. Furthermore, Mpayment has been widely accepted, due to both its contactless features and reliable performance and also to Chinese government restrictions established to prevent direct interaction and preserve social distancing during the C-19 outbreak. Users' sense of safety and positive attitudes when using Mpayment as their primary payment method have been shown to reduce the risk of virus transmission, ensure personal safety, and support the social economy [5]. Thus, contactless payment systems maintain social distancing as users make payments. In this context, social distancing can be defined as maintaining a safe distance between the merchant and buyer, without any need for either person to touch the payment terminal. The present study offers the following hypothesis, based on the aforementioned rationale:

H11 Social distancing has had a positive effect on the adoption of Mpayment during the coronavirus outbreak.

## 4 Methodology

To validate the proposed conceptual model and investigate the research hypothesis, an online survey (www.wenjuan.com/s/UZBZJvz9O7/#) was created to gather data via a popular Chinese social-media platform (WeChat). An online survey was considered the fastest and most efficient way to collect opinions on this topic, even though it excluded people who lacked immediate access to the Internet. The measurements were written in English and translated into Chinese by an expert, with the final set of measurements incorporating both languages, (English and Chinese). The questionnaire was divided into two sections. The first section presented demographic information (see Table 1), while the second (see Appendix A) comprised 31 items drawn from previous research and scored using a five-point Likert scale (with responses ranging from “strongly disagree” to “strongly agree”). In total, 593 complete and valid surveys were received for the statistical analysis; of these 322 (54.3%) were from men and 271 (45.7%) were from women. The sample size was large enough to provide accurate results for the entire study population.

**Table 1:** Demographic data.

Items	Types	Participants	
		N	%
Gender	Male	322	54.3
	Female	271	45.7
Age	Less than 20	134	23
	20–25	164	28
	26–30	120	20
	31–35	109	18
	36 and above	66	11
Income Range In RMB	1000–10000	443	75
	10001–20000	103	17
	20001–30000	38	0.6
	30000 and above	9	01
High Education Attainment	High School	124	21
	Bachelor	321	54
	Master	109	18
	PhD	39	06
Total		593	100

### 4.1 Exploratory and confirmatory factor analysis

A validity analysis was performed to evaluate the questionnaire data, with scales used to determine the degree of validity of each hypothesis [55]. There are many types of validity, including convergent and discriminant validity. Bartlett’s sphericity test and the Kaiser–Meyer–Olkin (KMO) test were used to assess the collected data. In Table 2, which shows the suitability of the factor analysis performed, the results are χ^2^ = 5013.937 (ρ = 0.000). Based on KMO measures, a value above 0.7 is acceptable, with higher validity levels closer to 1. As the KMO measure in this study is 0.803, the collected data have valid convergent validity; ρ = 0.000 indicates that the data are suitable for differentiation [56].

**Table 2:** KMO and Bartlett's test.

KMO	0.804
χ^2^	5013.937
df	567
ρ	0.000

To test the study model, researchers applied structural equation modelling (SEM). The partial least squares (PLS) [57] method of statistical analysis was chosen as an effective way to measure and analyze psychometric properties, reliability, and validity [57]. Assessment requires tests for the reliability and validity of each construct; Cronbach’s alpha was used to assess construct reliability. As Table 3 shows, all of the Cronbach's alpha values for latent constructs exceeded 0.70, which indicates construct consistency, according to Nunnally and Bernstein, [58,59]. The composite reliability (CR) was also tested and found to be higher than the 0.70 threshold (see Table 3), thereby supporting the model’s good reliability and internal consistency [58,60]. The average variance extracted (AVE) was used to verify convergent validity by calculating each construct item loading [59]. As Table 3 shows, the item loadings were all greater than 0.40, with AVE measurements greater than 0.50. The results of the factor correlation analysis are shown in Table 4.

**Table 3:** Descriptive statistics and psychometric properties.

	Item	M	SD	Loading	AVE	Cronbach's Alpha(α)	CR
PE	1	3.3120	1.03591	.908	0.801	.729	0.942
	2	2.7470	1.45661	.897			
	3	3.2934	1.10507	.894			
	4	2.7302	1.47320	.881			
EE	1	3.3727	1.03375	.789	0.529	.717	0.817
	2	3.4351	1.11785	.768			
	3	3.2125	1.13247	.725			
	4	3.4115	.94046	.615			
PT	1	3.2260	1.08407	.834	0.621	.715	0.868
	2	3.4435	1.05135	.814			
	3	3.3710	1.06573	.752			
	4	3.4536	1.04540	.749			
PS	1	3.4469	1.04022	.738	0.552	.742	0.831
	2	3.3019	1.05984	.787			
	3	3.3002	1.07692	.729			
	4	3.3676	.99480	.717			
SI	1	3.4081	1.02933	.753	0.534	.703	0.775
	2	2.7707	1.47089	.743			
	3	3.3406	1.03909	.696			
C-19 fear	1	3.3609	1.00738	.788	0.587	.784	0.851
	2	3.4654	1.02465	.785			
	3	3.3592	1.00882	.751			
	4	2.7470	1.45545	.741			
SD	1	3.3642	.99434	.756	0.553	.731	0.833
	2	3.3895	1.10970	.746			
	3	3.1096	1.07480	.741			
	4	2.8128	1.32046	.734			
BI	1	2.9781	1.36728	.787	0.561	.736	0.837
	2	3.0101	1.13597	.750			
	3	3.0961	1.06663	.735			
	4	3.3710	.98843	.725

**Table 4:** The correlation between factors.

	1	2	3	4	5	6	7	8
PE	–							
EE	.114**	–						
TR	.174**	.464**	–					
PS	.164**	.236**	.237**	–				
SI	.533**	.301**	.304**	.188**	–			
C-19	.520**	.353**	.420**	.199**	.532**	–		
SD	.185**	.321**	.268**	.190**	.195**	.288**	–	
BI	.185**	.253**	.200**	.183**	.228**	.265**	.245**	–

∗∗ Correlation is sig at 0.01.

### 4.2 Findings

**Fig. 2:**
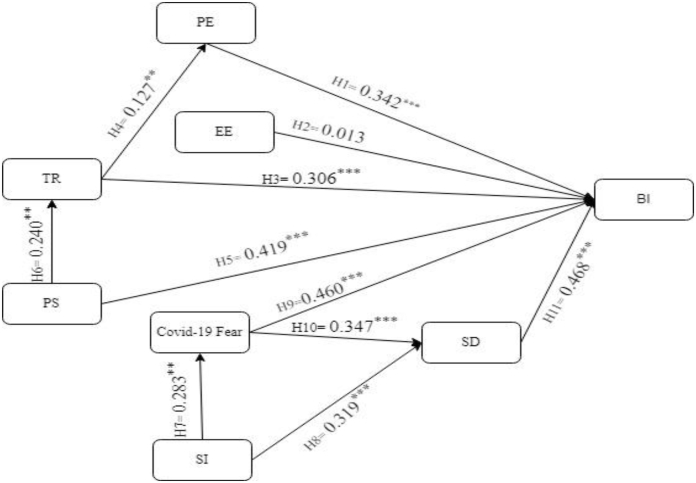
The results of the study model.

An exploratory factor analysis (EFA) was used to evaluate the data, regardless of whether the research components had theoretical justification. The primary goal of a confirmatory factor analysis (CFA) is to confirm that the model fits the obtained data and the components are loaded properly. Following the EFA, a CFA was performed to assess the hypothesized model associations between the variables. This analysis also examined the data and relationships to determine whether the model was appropriate and logical [61]. A path analysis was used to examine the relationships between factors in the hypothetical model and to discover which elements were taken into account by users [62]. The statistical findings are presented in Fig. 2 of the model and in Table 6.

**Table 5:** Model fit indices.

Models Fit	Criteria	Measurement Model
CMIN(X^2^)	–	433.634
DF	–	273
X^2^/DF	≤5	1.588
CFI	≥.90	0.913
GFI	≥.90	0.905
AGFI	≥.90	0.910
TLI	≥.90	0.912
NFI	≥.90	0.900
RMSEA	≤.08	0.051

**Table 6:** Correlational statistics for the study hypotheses.

Items	R	R^2^	p-value	Result
PE → BI	0.585***	0.342***	0.000	Accepted
EE → BI	0.112	0.013	0.321	Rejected
TR → BI	0.554***	0.306***	0.000	Accepted
TR → PE	0.356**	0.127**	0.014	Accepted
PS → BI	0.648***	0.419***	0.000	Accepted
PS→TR	0.490**	0.240**	0.012	Accepted
SI→Covid-19	0.532***	0.283**	0.010	Accepted
SI → SD	0.565***	0.319***	0.000	Accepted
Covid-19 → BI	0.678***	0.460***	0.000	Accepted
Covid-19 → SD	0.589***	0.347***	0.000	Accepted
SD → BI	0.684***	0.468***	0.000	Accepted

Next, a CFA was conducted to measure the model fit index. Many fit indices, such as the goodness-of-fit index, are used in SEM to evaluate an entire model [62]. The most essential fit index is X2, which serves as the basis for numerous other indices and can also be used to evaluate null hypotheses. In the present study, the indices in Table 5 were used to measure model fit. They show that the model does meet the criteria.

## 5 Discussion

Based on the data and findings, most study hypotheses were verified, confirming that the adoption model adequately explained the factors that influenced Mpayment-adoption intentions during the C-19 pandemic. Perceived expectancy (H:1) had a direct positive effect on the intention to use Mpayment during the C-19 pandemic, in line with previous findings [44,63]. The utility and operability of Mpayment technology were expected to boost users' payment competence during an emergency situation because Mpayments offered a speedy payment mechanism that required no direct or indirect connections between individuals. Throughout the pandemic, this feature had an important effect on the adoption intentions of users, who generally considered Mpayments a more beneficial and trustworthy way to make transactions than traditional payments. However, the findings do not support effort expectancy (H:2), indicating that the understanding and use of Mpayment systems had no direct influence on users' behavioral intention to accept Mpayments during the C-19 pandemic. Previous studies in the field of Mpayment research have produced similar results [12,64]. The key explanation for this finding is that users have become acclimated to smartphone operations and more skilled as a consequence of using numerous smartphone apps in the past [65]. During the C-19 pandemic, many conceptions of personal safety, including dependability, usefulness, security, trustworthiness, and incentives, had a stronger impact on user behavior, supporting a multifaceted case for maintaining payment transactions. However, Mpayment effort expectancy had a less significant or surmountable effect on consumer adoption intentions during the pandemic. This study also shows that trust (H:3) had a direct and significant impact on users' behavioral intention to use Mpayments during the C-19 pandemic, as well as an indirect effect (via H:4) that potentially explains their behavioral intention to use Mpayment during the C-19 pandemic. Specifically, consumers have developed an established sense of trust in Mpayment services, due to their consistent performance and mature legal-framework protection. As a consequence, they enjoy greater benefits from the service and are less concerned about financial risks [66]. Chinese citizens trust the Mpayment platform because it is government-regulated. A study conducted by Abubker et al. [67], confirms that government monitoring is the most important factor underpinning customer trust in payment acceptance in China. The C-19 pandemic added fear and social pressure to people's daily lives. The reliability of Mpayment systems has increased the likelihood that consumers will use them to make contactless Mpayments in place of conventional payments [30]. The participants' perception that cashless payments can protect them from the virus has made them more likely to choose Mpayments more frequently.

Furthermore, in the event of a coronavirus pandemic, perceived security (H:5) has a direct and significant beneficial influence on the desire to use Mpayments. During the C-19 pandemic, perceived security (H:6) was associated with trust among customers using Mpayments. User perceptions of security may reduce uncertainty while crucially ensuring Mpayment performance, thus building user trust in Mpayment systems [67]. There is a significant link between trust and perceived security; both traits made customers more willing to use Mpayments during the epidemic. Furthermore, because Mpayments involve personal data, it is essential to validate the trustworthiness and authenticity of Mpayment systems to secure transactions and protect personal data [68]. The security, truthfulness, and reliability of Mpayment systems encouraged users to consent to the collection of information about their payment times and locations during the pandemic. These data have been used by governments and health organizations to track connections among payment processes, as well as to monitor, update, and report on the status of pandemic transmissions. The information gleaned from Mpayments can make users aware of the spread of the virus in a clear and timely manner. This benefit may positively influence their decision to use Mpayments to limit the risk of C-19 infection.

The present study also shows that social influence (H:7) positively and significantly influences fear of C-19 and social distancing (H:8), encouraging more people to adopt Mpayment during the C-19 pandemic. During the pandemic, advice and recommendations from important and relevant people became even more vital in directing individuals to make decisions and take necessary actions. The fact that people were socially influenced by family members and close friends may have increased their fear of C-19. This fear reduced direct contact among people, decreased the wide spread of the coronavirus, and increased the adoption of Mpayments, due to their contactless features [69]. Mpayment was commonly recommended as a safe and useful payment solution by friends and family members because it minimized human participation throughout the transaction process. As a consequence, the reputation of Mpayment and its word-of-mouth (WOM) impact are seen as critical in promoting people’s Mpayment acceptance intentions and encouraging them to develop a new payment habit in response to the pandemic [44].

Overall, social distancing (H:11) had the most significant positive effect on Mpayment adoption during the coronavirus pandemic, followed by fear of C-19 (H:9). According to the present study, social distancing, fear of C-19, and the applicability of Mpayment technology can increase users' payment efficiency in emergency situations. The psychological processes by which users accepted Mpayments were highly impacted by their contactless nature, which was well suited to the prevailing environmental conditions, public restrictions, and individual needs during the C-19 crisis. This finding is in line with a previous study [44]. Moreover, fear of C-19 (H:10) has been found to have a significant positive association with social distancing, thus encouraging people to use Mpayments to maintain indirect (e.g, socially distanced) connections. One key concept in the current investigation is the fear caused by C-19 dissemination. The C-19 pandemic has significantly impacted human communities. Lockdown, social distancing, and staying at home are recommended methods of preventing transmission, [1,70]. In comparison to traditional payments, contactless Mpayments can help consumers maintain social distancing by eliminating direct and indirect encounters with currency or point-of-sale terminals throughout the transaction process. During the C-19 pandemic, this feature allowed people to express their opinions about personal safety and utility, while using Mpayment technology as a financial-transaction method.

### 5.1 Theoretical and practical implications

The findings of the present study have three major theoretical ramifications. First, this study is empirical in nature, investigating the factors that influence users' intention to adopt Mpayments during a pandemic situation, a focus that is missing from prior research. The present study significantly expands the literature on technology uptake during epidemics. In particular, it models a valuable way of understanding user adoption intentions by measuring user expectations and investigating user attitudes from a technological perspective.

This study also shows that emergency situations have a significant impact on consumer impressions of technology. During the pandemic, the practical applications of new technologies became more evident, as users relied on them to undertake remote communication and to facilitate distant study, work, and interpersonal connections. Such technologies have played a major role in minimizing the consequences of social-distancing restrictions. In fact, the adoption of new technological solutions may be a consequence of people recognizing their utility, rather than wanting to test them. The present study therefore paves the way for future researchers to assess new cases of technology adoption from the perspective of users facing specific conditions—in particular, emergency situations. Second, this study adds new components to the UTAUT model, including perceived security and trust, fear of C-19, and social distancing, thus helping to broaden the literature on IT adoption. Fourth, it focuses on investigating technological features that relate closely to pandemic conditions as prospective antecedents and potential variables, which influence users' psychological and technological perspectives and perceptions. In particular, the contactless nature of Mpayments prevents interaction during transactions and preserves social distance, providing multiple perceived advantages for consumers. At the same time, it strengthens their understanding of how and why to use Mpayments in the content of a pandemic. By contrast, the C-19 pandemic has made effort expectations less significant than other factors for users deciding whether to accept Mpayments.

The current study builds on previous research on Mpayment-adoption intentions in emergency circumstances by exploring how a global pandemic (and by extension, other types of epidemic) can affect customer payment habits. It confirms that any type of pandemic or epidemic can cause individuals and communities to suffer. At the same time, it promotes the creation of new technologies, capable of supporting people, companies, and societies during emergency circumstances, while helping key stakeholders consider ways to design suitable business strategies to protect communities against future pandemics and epidemics. This study may also prove useful to new Mpayment service operators, regulators, and government entities. Mobile payments are gaining in popularity and providing significant services that enable quick transactions, especially during emergencies. During a pandemic or epidemic, Mpayments can boost personal safety and company stability. The findings of the present study can help stakeholders recognize the relevance of Mpayments in providing perceived advantages to users and developing appropriate system features. To fulfill consumer needs and lifestyles, mobile-payment service providers should provide operational compatibility, efficiency, and security. Enhancing public perceptions of Mpayments and encouraging beneficial social effects will boost the reputations of technology providers in many situations.

A further benefit of this research is the fact that it offers new technology developers a comprehensive understanding of customer adoption intentions, which are impacted by both technological and psychological considerations. It shows that service providers should concentrate on mobile contactless-payment features, which eliminate direct or indirect contact and reduce disease transmission, thus increasing adoption in the target audience. Finally, the results and outcomes of this study may be used as models for other service businesses in the event of a future epidemic or pandemic.

### 5.2 Limitations and future research

This study has some limitations. First, the data were collected online, leading people unable to use or access the Internet to be underrepresented. Since the data were collected in China during the C-19 pandemic, the findings may not be generalizable to other countries or conditions. Future studies should follow a similar strategy, collecting data from a wide range of countries and considering the distinct benefits associated with particular situations. Cross-cultural studies can be used to evaluate the research model and gain a better understanding of variations across cultures. Second, this study focuses on a few factors, mainly associated with technology acceptance. Future research should investigate more technological and psychological factors to gain a deeper understanding of the elements that influence the acceptance of new technology.

In addition to potential applications related to COVID-19, this study can be used to inform the implementation of other technologies in other areas, such as healthcare and education. The model can help researchers design strategies that consider the technological and psychological elements that impact users when new technologies are introduced. These findings can also help stakeholders develop effective strategies to increase the usability of new technologies, reduce user frustration, and improve the overall user experience.

## 6 Conclusions

The C-19 pandemic caused a catastrophic situation that significantly affected customer payment habits. In doing so, it created an opportunity to improve consumer acceptance of cashless transactions by amplifying the impact of factors that were previously undervalued in customer decisions and payment-preference studies. The present study model demonstrates considerable explanatory power when describing how consumer payment habits altered as a result of the pandemic and how Mpayment-adoption intentions were impacted by users' psychological and technological perspectives. In particular, the contactless nature of Mpayment systems proved highly advantageous in preserving social distance and ensuring personal safety during the pandemic. As the present study shows, two factors (social distancing and fear of C-19) had the greatest impact on users' Mpayment adoption intentions. In addition, performance expectancy, perceived trust, perceived security, and social influence all had a significant impact on Mpayment uptake during the C-19 pandemic. Interestingly, effort expectancy was found to have a negative impact on Mpayment adoption. The present study makes several significant theoretical and practical contributions to the literature on novel-technology acceptance in specific situations, advancing our knowledge and understanding of UTAUT extension by demonstrating, first, how user payment habits changed as a result of the pandemic, and second, how users' psychological and technological perceptions affected their intention to adopt Mpayments. As Mpayments are contactless, this study recommends that the government continue to encourage individuals to use them. A fuller understanding of user behavior will allow for an effective analysis of new-technology adoption and the development of strategies that optimize user experiences.

### Author contribution statement

Kamal Abubker Abrahim Sleiman: wang Yubo: Wenge Rong: Lan Juanli: Hongzhen Lei: Shuanhang Li: Jingyi Cheng: Fouzia Amin: Conceived and designed the experiments; Performed the experiments; Analyzed and interpreted the data; Contributed reagents, materials, analysis tools or data; Wrote the paper. (please add the names as its appear in the first page of the title)

## Data availability statement

Data will be made available on request.

## Declaration of competing interest

The authors declare that they have no known competing financial interests or personal relationships that could have appeared to influence the work reported in this paper

## Figures and Tables

**Table:** 

Factors	Items	References
	Performance expectancy (PE)	[32]
PE1	I believe that mobile payment is a good method of making purchases, during the coronavirus outbreak	
PE2	Adopting mobile payment makes the processing of transactions during the coronavirus outbreak much easier to manage	
PE3	Adopting mobile payment increases the effectiveness of my payment processing during the coronavirus outbreak	
PE4	Using mobile payment during coronavirus outbreak, allows me to receive my payment more swiftly.	
	Effort expectancy (EE)	[2,83]
EE1	It's easy to master how to employ mobile payment	
EE2	It is simple to complete all of the mobile payment steps	
EE3	It is effortless for me to be expert in the use of mobile payment	
EE4	The interaction with mobile payment is straightforward and understandable during the coronavirus epidemic	
	Trust (TR)	[8,67]
TR1	I believe mobile payment systems are competent and effective in processing my contactless payments during the coronavirus epidemic	
TR2	During the coronavirus epidemic, I expect that the mobile payment platform will prioritize user interests.	
TR3	I believe mobile payments are trustworthy, During the coronavirus epidemic.	
TR4	I believe mobile payment systems are dependable, during the coronavirus epidemic	
	Perceived security (PS)	[67,71]
PS1	I feel safe using my mobile payments, during the coronavirus epidemic	
PS2	I would consider mobile payment services to be secure, When making financial transactions during the coronavirus epidemic.	
PS3	When using mobile payments during the coronavirus epidemic, I felt safe providing personal information.	
PS4	Mobile payment systems have a minimal chance of billing information being misused.	
	Social influence (SI)	[72]
SI1	People close to me have advised me to utilize mobile payments, during the coronavirus epidemic.	
SI2	People who are close to me believe that, amid a coronavirus outbreak, mobile payment is necessary to me.	
SI3	People close to me are boosting me to use mobile payments during the coronavirus epidemic	
	Coronavirus fear (C-19)	[69]
C-191	Since the coronavirus epidemic, I've avoided paying with cash.	
C-192	Because of the coronavirus epidemic, I'm fearful of socializing with other people.	
C-193	Mobile payment reduces my fear	
C-194	I am afraid of losing my life due to coronavirus epidemic	
	Social distancing (SD)	[1]
SD1	I anticipate to be able to pay for anything without using currency, Because of the coronavirus epidemic	
SD2	Because of the coronavirus outbreak I'm interested in accepting payments without touching the terminal	
SD3	Because of the coronavirus outbreak, I'm beginning to feel that payment systems protect me from the virus	
SD4	Because of the coronavirus epidemic, I'm more aware of the general benefit of modern technology	
	Behavioral intention (BI)	[32]
BI1	I intend to employ mobile payments during the coronavirus epidemic	
BI2	I am willing to utilize mobile payments on a regular basis, during the coronavirus epidemic	
BI3	I am willing to use mobile payment as my primary payment option in various transaction procedures.	
BI4	I intent to continuously use mobile payments in the future	
